# Peroneal artery pseudoaneurysm with arteriovenous fistula after calcaneal fracture surgery: a case report

**DOI:** 10.3389/fsurg.2025.1527992

**Published:** 2025-02-17

**Authors:** Yu-Long Li, Sheng Chen, Yu Ai, Wei Zhang, Ji-Chao Liu, Ya-Fan Yang

**Affiliations:** Department of Orthopaedics, Xiantao First People’s Hospital, Xiantao, Hubei, China

**Keywords:** peroneal artery, delayed pseudoaneurysm, arteriovenous fistula, calcaneal fracture, arterial injury, case report, tarsal sinus incision

## Abstract

**Background:**

Peroneal artery injury following internal fixation of a calcaneal fracture is rare. The occurrence of a delayed pseudoaneurysm with an arteriovenous fistula after a peroneal artery injury is even rarer and has not been reported previously.

**Case summary:**

Herein, we report the case of a 65-year-old female patient with a calcaneal fracture (Sanders Type ⅢAB) who underwent an open reduction and internal fixation surgery through the tarsal sinus approach. Two months postoperatively, she experienced left foot pain. Physical examination revealed pulsation at the surgical site and a positive Branham sign, which was suggestive of a delayed pseudoaneurysm. Emergency digital subtraction angiography (DSA) examination was performed, and this revealed a pseudoaneurysm and arteriovenous fistula at the distal end of the peroneal artery. Therefore, the patient underwent transcatheter coil embolization of the peroneal artery and had good postoperative outcomes.

**Conclusion:**

Arterial injury should be suspected if massive hemorrhage occurs shortly after internal fixation for calcaneal fractures, and appropriate hemostatic measures should be promptly instituted. If unexplained pain and swelling develop along with a palpable pulse at the surgical site, a pseudoaneurysm should be suspected, and appropriate examinations should be promptly performed. Accurate diagnosis and prompt treatment are also crucial.

## 1 Introduction

Peroneal artery injury after internal fixation of a calcaneal fracture is an uncommon clinical entity. A delayed pseudoaneurysm with an arteriovenous fistula after a peroneal artery injury is even rarer (1).

The tarsal sinus incision for the treatment of calcaneal fractures is an important cause of peroneal artery end injury, the failure of hemostasis and the ongoing bleeding may lead to the formation of a pseudoaneurysm and an arteriovenous fistula (2).

Herein, we report the case of a patient who developed a pseudoaneurysm of the peroneal artery complicated by an arteriovenous fistula after surgical treatment of a calcaneal fracture through a tarsal sinus incision. In this report, we also discuss the causes and therapeutic options of this rare entity.

## 2 Case presentation

### 2.1 Clinical findings

A 65-year-old woman presented with a 3 h history of left foot pain due to a left calcaneal fracture secondary to a fall.

She had a history of hypertension that was diagnosed in February 2009 and was treated with nifedipine sustained-release tablets.

On physical examination, the patient's vital signs were as follows: Blood pressure, 185/110 mmHg. Furthermore, the patient had left foot swelling, tenderness, and heel varus deformity. She had no open wounds, no sensory or motor disorders, and good peripheral circulation.

**Figure 1:**
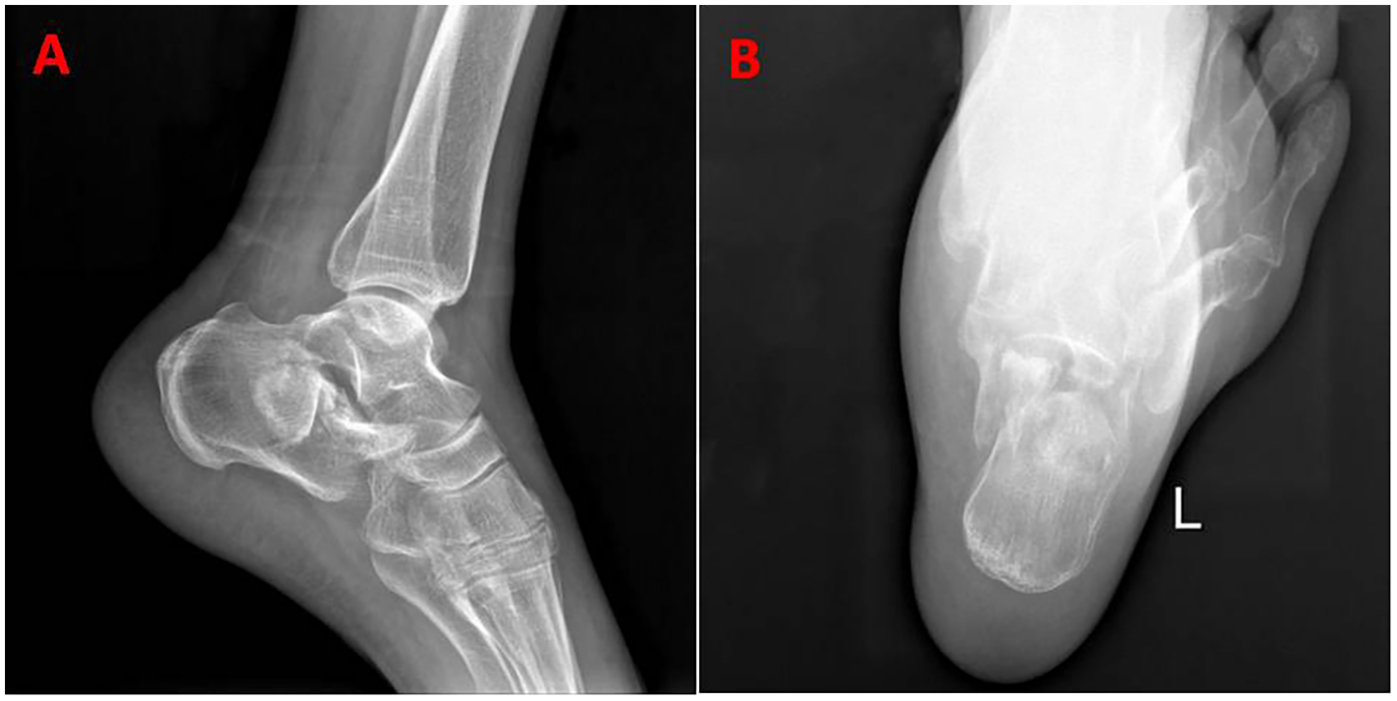
**(A,B)** Preoperative x-ray of the left foot shows a calcaneal fracture.

Laboratory tests revealed the following: prothrombin activity, 59% (80%–100%); prothrombin time, 17.3 s(9.4–12.5 s); international normalized ratio, 1.40(0.85–1.15); partial thromboplastin time, 45.9 s(25.1–36.5 s), fibrinogen, 1.43 g/L(2–4 g/L); total bilirubin, 37.8 umol/L(0–24 umol/L); direct bilirubin, 15.9 umol/L(0–8 umol/L); albumin, 24.4 g/L(40–55 g/L); AST, 84 IU/L(15–40 IU/L); ALT, 75 IU/L(9–50 IU/L); and hepatitis C antibody, 682.07 s/co(0–1 s/co). No abnormalities were found on routine blood and urine tests. A radiograph of the patient's left foot revealed a clear fracture line on the left calcaneus with significant bone displacement of the posterior talocalcaneal joint (Figure 1).

### 2.2 Final diagnosis

After considering the patient's medical history and laboratory findings, the following diagnoses were made: left calcaneal fracture (Sanders type IIIAB), hepatitis C, abnormal coagulation function, impaired liver function, and third stage hypertension.

### 2.3 Treatment

As the calcaneal fracture classification was Sanders type IIIAB, we should restore the Böhler's angle (3). To get better outcoms, we prepared to treat it with ORIF (3). The patient underwent open reduction and internal fixation of the left calcaneal fracture through a tarsal sinus incision 11 days after being admitted when the foot swelling subsides. The fracture was adequately reduced and fixed, and a drainage tube was left *in situ*. The surgical incision was then sutured and dressed. One day postoperatively, the blood pressure was 170/105 mmHg, the drainage tube was removed, and pressure bandaging was continued. In the afternoon of the same day, the patient had a massive hemorrhage from the surgical site, which was controlled using pressure bandaging. The patient's hemoglobin level was decreased and her coagulation function was abnormal, as the hemorrhage had stopped, we didn't performed an additional investigation with echocolor Doppler or CT angiography, so that we failed to detect the injury of the peroneal artery in its early stage; therefore, she was transfused with packed red blood cells, fresh frozen plasma, and cryoprecipitate. Antiviral treatment for Hepatitis C was also administered. Although the surgical incision had healed 2 weeks postoperatively, a subcutaneous hematoma was still observed at the surgical site.

**Figure 2:**
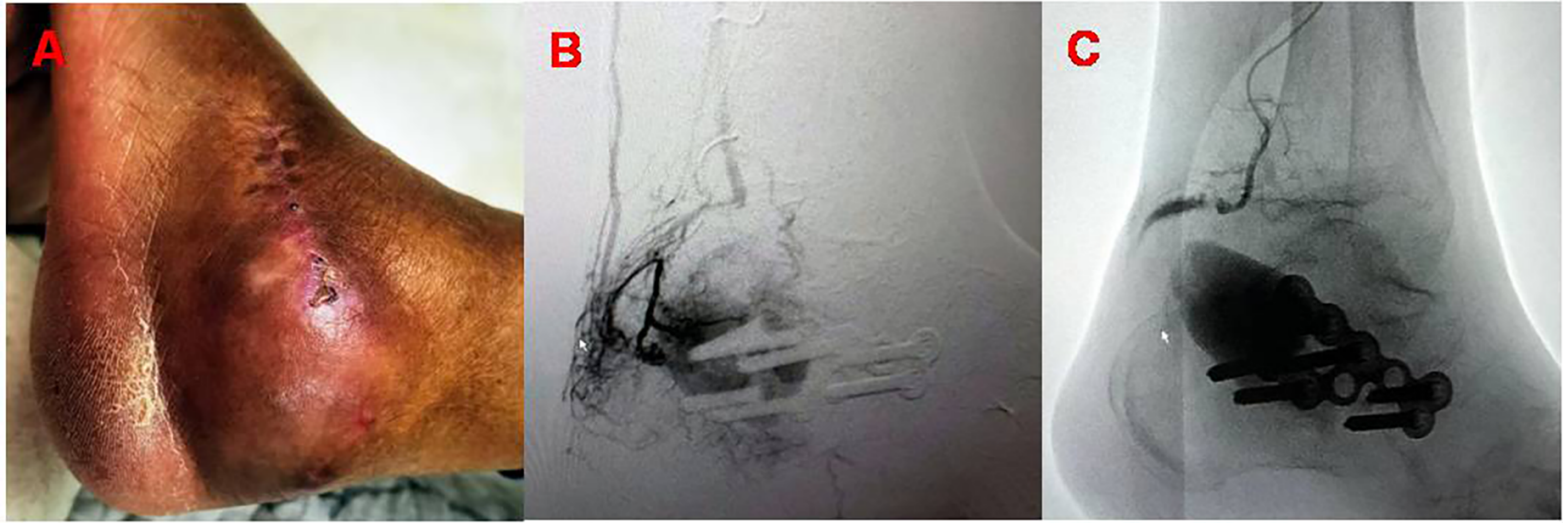
**(A)** Appearance of the left foot at the 2-month postoperative follow-up visit; **(B)** DSA imaging reveals features consistent with the formation of a peroneal artery pseudoaneurysm and arteriovenous fistula; **(C)** imaging after spring microcoil embolization of the peroneal artery pseudoaneurysm and arteriovenous fistula.

Two months after surgery, the patient still felt pain in her left foot and was admitted to the hospital. Physical examination revealed a palpable pulse at the surgical site and a positive Branham sign. Fresh blood was also aspirated through a puncture at the surgical site. Therefore, a pseudoaneurysm complicated by an arteriovenous fistula was suspected. Considering that DSA is the gold standard for the diagnosis of pseudoaneurysms, we performed the DSA examination directly. A Digital Subtraction Angiography (DSA) of the lower limb blood vessels revealed significant tortuosity at the distal end of the peroneal artery, rupture of the peroneal artery branches with a large amount of contrast agent spilling out and filling a sac-like structure on the lateral side of the left calcaneus, and numerous arteriovenous fistulas around it, with both ends of the carrying artery supplying blood. This confirmed the formation of a peroneal artery pseudoaneurysm complicated by an arteriovenous fistula. The patient underwent transcatheter coil embolization of the peroneal artery and had good postoperative outcomes. The patient was discharged the following day (Figure 2).

### 2.4 Outcome and follow-up

**Figure 3:**
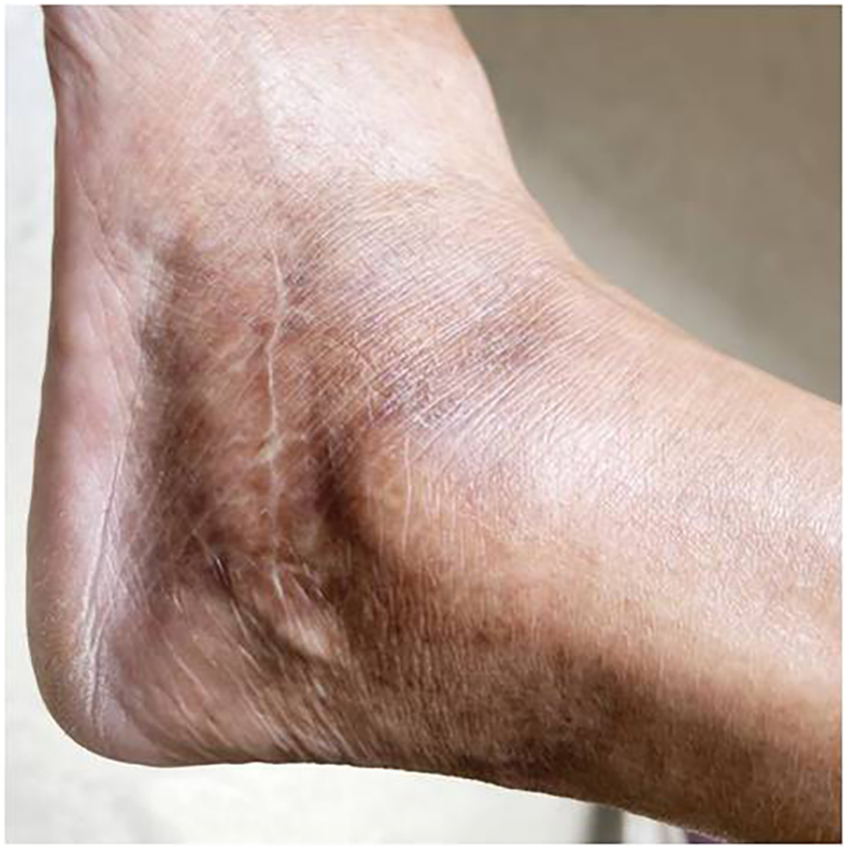
Appearance of the left foot at the 3-year postoperative follow-up visit.

After 3 years of follow-up, the patient reported no pain, swelling, or subcutaneous pulsation in her left foot (Figure 3).

## 3 Discussion

Aneurysms are pathologically classified as real aneurysms, pseudoaneurysms, and dissecting aneurysms. Pseudoaneurysms are mostly caused by trauma, which can be iatrogenic or non-iatrogenic (4). When blood vessels are damaged, blood flows into the tissue spaces and forms a hematoma, which in turn develops a fibrous capsule wall and is formed into a pseudoaneurysm. Pseudoaneurysm accounts for approximately 1% of all aneurysms (5). Arteriovenous fistulas are abnormal rapid channels between arteries and veins, and can be caused by aneurysms, infections, and vascular injuries. Hence, arterial vessel injuries can lead to the formation of pseudoaneurysms and arteriovenous fistulas.

Several factors can lead to arterial injuries in orthopedic surgery, including direct puncture of the fracture block, intraoperative injury, puncture injury of the drainage tube, and improper placement of screws and other internal fixation devices. The peroneal artery is a branch of the posterior tibial artery that runs obliquely behind theupper part of the flexor hallucis longus muscle, descends between the posterior part of thefibula and the tibialis posterior muscle, and distally accompanies the sural nerve. The tarsal sinus approach is used for the management of calcaneal fractures. During the procedure, the surgical incision is extended from 1 cm below the tip of the lateral malleolus to the distal 4th metatarsal, which is not generally associated with damage to the peroneal artery. However, because the steel plate is placed at the distal end, the incision needs to be extended to the Achilles tendon, which increases the risk of peroneal artery injury. Additionally, during the operation, the surface of the calcaneus needs to be sharply separated using a surgical blade; however, it cannot be separated under direct vision, which increases the possibility of peroneal artery injury. Based on the above factors, the treatment of calcaneal fractures using a tarsal sinus incision is an important cause of peroneal artery end injury. The peroneal artery thins into an arteriole, especially at its terminal part; hence, the blood pressure at this part is not high. After injury, a thrombus forms in the blood vessel to stop bleeding through local compression (6, 7). On the first day after the operation, the patient had a massive hemorrhage from the surgical site, which was controlled using pressure bandaging. The surgical incision healed at a later time. However, the peroneal artery did not thrombose and continued to bleed, resulting in a pseudoaneurysm. This was likely caused by the patient's poor coagulation function and high blood pressure. Due to our failure to detect the pseudoaneurysm in a timely manner, it led to the formation of an arteriovenous fistula.

After an arterial wall injury, a pseudoaneurysm can develop within hours to months (6, 8). The clinical manifestations of delayed pseudoaneurysms are limb pain, swelling, and progressive anemia (6, 7, 9). Physical examination can reveal a local mass, tenderness, and pulsation under the skin. When an arteriovenous fistula is formed, local tremor can be palpated at the site, and a rough, persistent, vascular murmur can be heard on auscultation along with a positive Branham sign. Imaging examinations should be performed when a pseudoaneurysm is suspected (10, 11). DSA is the gold standard for the diagnosis of pseudoaneurysms, as it can clarify the relationship between an artery and an aneurysm, and also reveal the hemodynamic features of the pathologies, while providing the basis for making appropriate treatment plans. Currently, the commonly used treatment methods for arteriovenous fistulas are thrombin injection under ultrasound guidance, transcatheter coil embolization under DSA, catheter embolization and surgical treatment (12–14). The patient in this report was treated with transcatheter coil embolization under DSA, as it was considered that DSA is more minimally invasive. and it had satisfactory postoperative outcomes.

## 4 Conclusion

The findings of this case report and a brief literature review suggest that a delayed pseudoaneurysm of the peroneal artery complicated by an arteriovenous fistula after calcaneal fracture is a very rare surgical complication that can be caused by iatrogenic injury and other comorbidities such as hypertension and coagulopathies. Therefore, adequate preparations should be made preoperatively, while identifying and promptly managing the patient's comorbidities. Surgeons should also be familiar with the relevant anatomy of the foot and operate carefully to avoid damaging the peroneal artery. And we can look for less traumatic techniques, including closed reduction, external fixation, and percutaneous surgery (PS), in order to reduce complication rate (15). If a vascular injury occurs postoperatively, it should be examined and treated promptly. Using these measures, the incidence of iatrogenic pseudoaneurysms complicated by arteriovenous fistulas can be reduced, and additional injury to patients can be avoided.

## Data Availability

The original contributions presented in the study are included in the article/Supplementary Material, further inquiries can be directed to the corresponding author.
